# New Insights into Cardiovascular and Exercise Physiology: A Compendium of the Special Issue

**DOI:** 10.3390/life15020252

**Published:** 2025-02-07

**Authors:** Helena Lenasi, Ines Drenjančević

**Affiliations:** 1 Institute of Physiology, Faculty of Medicine, University of Ljubljana, 1000 Ljubljana, Slovenia; 2 Department of Physiology and Immunology, Faculty of Medicine Osijek, University Josip Juraj Strossmayer, 31000 Osijek, Croatia; 3 Scientific Centre of Excellence for Personalized Health Care University Josip Juraj Strossmayer, 31000 Osijek, Croatia

## 1. Introduction

The capacity of the cardiovascular system to adjust to varying needs is immense. This Special Issue aims to present recent knowledge in cardiovascular and exercise physiology, and address some clinical implications.

Considering that the incidence of cardiovascular diseases is increasing, and that cardiovascular diseases present a major cause of death worldwide, the need to accomplish treatment strategies is also increasing. The knowledge of the physiological processes behind them remains a prerequisite for upgraded clinical studies.

Although many new mechanisms on the micro- and macro-scales have been revealed in recent years, which is also due to the evolution of modern techniques and new concepts that are emerging almost daily, there are still many controversies and enigmas. The never-ending challenge remains how to translate the findings obtained in cell cultures and studies using animal models into human physiology. In this respect, in vivo studies on humans are encouraged, as they reflect the real situation, and could strongly support clinical work.

Physical exercise has been increasingly regarded as one of the potentially beneficial measures to improve cardiovascular health, interfering with numerous elements of the cardiovascular system and implying a multitude of potential mechanisms [1]. Its long-term beneficial effects are well known; nevertheless, many questions remain unresolved. Where is the line between benefit and harm? How do different types of exercise affect the cardiovascular system in health and disease? What is the most appropriate measure regarding duration, repetition, and recovery, and whether and how exercise regimes can be individually adjusted? In addition, a proper evaluation of the physical parameters and cardio-pulmonary and training status are crucial, both in the light of the athlete’s achievements and improvements, and from the clinical point of view.

In this Special Issue, scientists from various fields of cardiovascular and/or sports medicine have presented their new findings: all studies were performed in humans, from investigations obtained in healthy individuals, ranging throughout the lifespan, from newborns to elderly, as well as in patients. The compilation encompasses eight original articles, two review papers, and two case reports, which are presented below, first presenting papers dealing with healthy persons, and later focusing on patients.

## 2. An Overview of the Published Articles

To start with the study involving the youngest participants, Lenasi et al. investigated the effects of caffeine in preterm newborns [2]. Caffeine, a central nervous system (CNS) stimulant of the methylxanthine class, exerts many complex and pleiotropic physiological effects on various organ systems. In newborns, it has empirically been used to treat apnea [3]. However, the study by Lenasi et al. aimed to assess whether it affects the maturation of the autonomic nervous system (ANS) in preterm newborns by analyzing their heart rate variability (HRV). Although caffeine increased breathing frequency (BF), it did not affect arterial oxygen saturation, body temperature, or the heart rate during sleeping in a supine position or any other HRV parameters. It appears that the maintenance dose of caffeine is too low to affect the heart rate and HRV. This study challenges further research to establish the exact mechanisms of caffeine’s multiple beneficial effects. Moreover, it exposes HRV as a useful tool for assessing the activity of the ANS in human studies.

Another study that also applied HRV as a measure of ANS activity was the study by Šorli and Lenasi [4], which elucidated the potential impacts of acute hyperglycemia induced by a standard oral glucose loading test on the microvascular reactivity at the time point of the peak plasma glucose concentration in young, healthy participants. Acute effects of hyperglycemia on microcirculation remain controversial in healthy individuals; decreased endothelial functions have been implicated, likely due to increased oxidative stress, decreased endothelial nitric oxide synthase (eNOS) activity, and the reduced bioavailability of vasodilator nitric oxide (NO) [5]. As insulin was also shown to interfere with endothelial signaling pathways, how these (to some extent) contradictory mechanisms interact at the vascular level in vivo is debatable. The study showed significant correlations between some post-occlusive reactive hyperemia (PORH) parameters, HRV parameters, and the plasma glucose concentration after glucose load, implying diminished vascular reactivity evoked by hyperglycemia in healthy subjects with lower glucose tolerance. Accordingly, this study exposes the need to develop stronger tools/markers for the timely detection of predisposed individuals.

Microvascular reactivity is mediated by endothelium-dependent mechanisms, which could be modified by exercise, but also by various external influences, such as a diet, related to a decrease in oxidative stress and an increase in antioxidative mechanisms. In athletes, although acute strenuous exercise leads to acute endothelial dysfunction, exercise increases their microvascular responsiveness, or leads to better utilization of vasodilatory capacity, consistent with the hormesis hypothesis. Dietary intake of n-3 polyunsaturated fatty acids [6] and antioxidant-enriched food has a beneficial effect on vasodilation. The results of the study of Kolar et al. provide evidence that athletes should enrich their diet with natural sources of micronutrients in the form of functional foods [7].

The next studies addressed the cardiopulmonary exercise testing (CPET). The study by Jurov et al. [8] sought an optimal model to estimate maximal oxygen consumption (VO_2max_) in trained cyclists. The general prediction equations applicable in nontrained individuals may underestimate the real VO_2max_ in trained persons. This study, conducted on 496 male and 84 female competitive cyclists, included six predictors: power output, body weight, body height, fat mass, fat-free mass, and age, and compared its accuracy to the traditional American College of Sports Medicine (ACSM) equation [9]. Three new equations were finally derived; power output and body weight were shown to be the most important parameters for predicting VO_2max_. This study demonstrates that the traditional ACSM equation strongly underestimates VO_2max_, exposing the need to evaluate prediction models for other athletes with a special focus on disciplines that demand high aerobic capacity.

Another valuable test used for monitoring sports performance and addressing the effectiveness of different training programs and neuromuscular fatigue is the countermovement jump (CMJ) with force platform. This device is considered the gold standard testing device, yielding different variable outputs [10]. The reliability of the CMJ-derived variables is greatly affected by the complexity of the computational methods: more computation renders less reliability. The study by Aničić et al. [11] aimed to assemble the list of necessary, highly reliable metrics derived from CMJ, and showed that only 24 out of 45 CMJ-derived variables had an acceptable reliability. The most reliable variables were performance variables, followed by kinetic variables, and finally kinematic variables. These variables were included in the principal component analysis, and loaded a total of four factors, explaining 91% of the CMJ variance: the performance component (variables responsible for overall jump performance), eccentric component (variables related to the breaking phase), concentric component (variables related to the upward phase), and jump strategy component (variables influencing the jumping style). This study revealed important implications for sports scientists and practitioners regarding the CMJ-derived metrics.

The study by Zimmermann et al. [12] aimed to define the physiological differences between two uphill locomotion patterns, namely uphill running versus uphill walking, in trail running (TR) athletes. The obtained data of the TR athletes were compared for anthropometric data and CPET parameters, such as maximal ventilation (*V*˙*E*_*max*_), VO_2max_, maximal BF (BF_max_), peak oxygen pulse, and the energy cost of running (C_r_). All TR athletes showed excellent performance data, whereby across both different uphill locomotion strategies, significant differences were revealed solely for *V*˙*E*_*max*_ and time to reach the mountain peak. These results provide new insights, and might contribute to a comprehensive understanding of cardiorespiratory consequences of short uphill locomotion strategy in TR athletes.

The next study, by Jurov et al. [13], is a meta-analysis investigating associations between VO_2max_ and body mass, year of the study, and country of origin of healthy prepubertal boys (mean age under 11 years old). Study aimed to extract new reference values for cardiorespiratory fitness, considering 95 study samples. The results show that the absolute VO_2max_ (Lmin^−1^) is higher in more recent studies, whereas the mean relative VO_2max_ is lower, stressing the important impact of body mass. Aerobic capacity normalized to body weight does not change with age. Cardiorespiratory fitness (CRF) in prepubertal boys is declining, associated with an increasing body mass over the last few decades. The study stresses the importance of distinguishing between the VO_2max_ and peak oxygen consumption (VO_2peak_) indicators. Given the lack of age- and gender-specific CRF reference values in prepubertal children, the study finally exposes a need to develop observed distributions of VO_2max_ based on criterion methods rather than estimated or regression-based predicted values and to compare the VO_2max_ between prepubertal boys and girls.

In addition to healthy individuals, exercise testing is a key in the risk stratification of patients with heart failure (HF). The study of Bras et al. [14] aimed to assess the predictive value of the heart transplantation (HTx) thresholds in HF in women and men by performing a prospective evaluation of HF patients who underwent CPET from 2009 to 2018 for the composite endpoint of cardiovascular mortality and urgent HTx. A total of 458 patients underwent CPET, with a composite endpoint frequency of 10.5% in females vs. 16.0% in males in the 36-month follow-up. According to the 2016 International Society for Heart Lung Transplantation (ISHLT) listing criteria for HTx, the recommended thresholds for pVO_2_ (≤12 mL/kg/min, or ≤14 mL/kg/min if intolerant to β-blockers), minute ventilation–CO_2_ production relationship (VE/VCO_2_ slope > 35), and percent of predicted VO_2peak_ ≤ 55%, showed a significantly higher overall diagnostic effectiveness in women compared to men [15]. Moreover, specific VO_2peak_, VE/VCO_2_ slope, and percent of predicted VO_2peak_ cut-offs in each sex group presented a higher prognostic power than the recommended thresholds. The authors concluded that individualized sex-specific thresholds may improve patient selection for HTx.

The narrative review by Škafar et al. [16] evaluated exercise stress echocardiography (ESE), a non-invasive, inexpensive, and widely available imaging modality for estimating systolic pulmonary arterial pressure (sPAP) as a method for diagnosis of pulmonary hypertension (PH), which is associated with numerous respiratory and/or cardiovascular diseases, and diagnosed by right heart catheterization. The newest ESC Guidelines for the diagnosis and treatment of PH reintroduced the diagnosis of the exercise PH [17]. ESE can distinguish between noncardiac and cardiac causes of unexplained dyspnea, and is useful in patients with connective tissue disease. However, there is a lack of validated criteria and prospective clinical studies. The paper reviews pulmonary hemodynamic responses to exercise, briefly describes the modalities for assessing pulmonary hemodynamics, and discusses contemporary key clinical applications of ESE in patients with PH.

The study by Michou et al. [18] also addressed patients. Increased physical activity is highly recommended in patients with diabetes mellitus and one of its complications, namely chronic kidney disease. Cardiac autonomic neuropathy is a complication of diabetes, and may contribute to increased risks. Since many diabetic patients with chronic kidney disease require hemodialysis, maintaining an exercise routine might be demanding for them. Thus, home-based exercise programs may help improve their overall fitness and cardiac functioning. The randomized controlled study assessed the effects of a 6-month home-based exercise training program consisting of three combined (aerobic and strengthening) exercise sessions per week on the non-dialysis days. The results demonstrated increased levels of HDL and a decrease in HbA1c, improved cardiovascular fitness indices, including decreased heart rate and systolic blood pressure, and favorable effects on sympathetic and vagal nerve activity and the sympathovagal balance.

The two last studies, by Ušaj et al. and Srdanović et al., respectively, are case reports.

Ušaj et al. [19] traced a single runner with a VO_2peak_ 74 mL∙min^−1^∙kg^−1^ over a preparatory period of two months (Everesting) and the subsequent one-month recovery period, which is a known period of reduced performance. During the first phase of the recovery, enhanced peak creatine kinase (800%) and C-reactive protein (44%) levels explained the decreased performance. In contrast, decreased performance during the second, longer phase was associated with a decreased lactate threshold and VO_2_ (21% and 17%, respectively), as well as an increased energetic cost of running (15%) and higher endogenous carbohydrate oxidation rates (87%), lactate concentrations (170%), and respiratory muscle fatigue sensations that remained elevated for up to one month. These alterations may represent the characteristics of the second phase of the recovery process after Everesting.

Takotsubo Cardiomyopathy (TCM) is a reversible cardiomyopathy characterized by transient regional systolic dysfunction of the left ventricle. It can clinically mimic myocardial infarction without obstruction of coronary arteries; however, it presents as a distinct condition. Interestingly, TCM can be concomitant to acute myocardial infarction with ST elevation (STEMI), which is the case in 5–8% of women with STEMI. Srdanović et al. [20] presented a 78-year-old woman who experienced simultaneous TCM and STEMI, which was successfully resolved with intensive medical care. This case showed that concomitant TCM and STEMI can lead to cardiogenic shock, which makes treatment challenging.

## 3. Concluding Remarks

This compilation of articles encompasses a diverse range of research, aspects, and approaches to exercise, with the common denominator being that they were all performed in humans, including healthy participants with differences in cardiopulmonary status and patients. This Special Issue stresses the importance of exercise as a daily routine, also appropriate as home-based exercises which might postpone, or at least ameliorate, disease progression. Overall, the findings reveal important implications for sports scientists, practitioners, and clinicians, stressing the importance of adjusted and proper exercise testing in healthy and in patients. Many manuscripts support the need for gender stratifications when adjusting either exercise testing or exercise regimes or evaluating a cohort of healthy participants and patients. In addition, the articles expose a need for standardization of various diagnostic tools in sports medicine and standardization of the evaluated parameters. In the future, special emphasis should be given to establishing the cut-off/predicted/recommended values of VO_2max_ in children, athletes, and patients, adjusted to the particular diseases. Since cardiovascular physiology is a huge field, encompassing many complex players and mechanisms, this Special Issue also exposes some unresolved questions, and encourages further research in this vast and complex field.

We believe this Special Issue would attract readers, with each manuscript included in this Special Issue uniquely contributing a puzzle to the whole mosaic and opening challenges and questions for future research.
